# Metal Binding Is Critical for the Folding and Function of Laminin Binding Protein, Lmb of *Streptococcus agalactiae*

**DOI:** 10.1371/journal.pone.0067517

**Published:** 2013-06-24

**Authors:** Preethi Ragunathan, Divya Sridaran, Anja Weigel, Sarah Shabayek, Barbara Spellerberg, Karthe Ponnuraj

**Affiliations:** 1 Centre of Advanced Study in Crystallography and Biophysics, University of Madras, Guindy Campus, Chennai, India; 2 Institute of Medical Microbiology and Hygiene, University of Ulm, Ulm, Germany; Research Center Borstel, Germany

## Abstract

Lmb is a 34 kDa laminin binding surface adhesin of *Streptococcus agalactiae*. The structure of Lmb reported by us recently has shown that it consists of a metal binding crevice, in which a zinc ion is coordinated to three highly conserved histidines. To elucidate the structural and functional significance of the metal ion in Lmb, these histidines have been mutated to alanine and single, double and triple mutants were generated. These mutations resulted in insolubility of the protein and revealed altered secondary and tertiary structures, as evidenced by circular dichroism and fluorescence spectroscopy studies. The mutations also significantly decreased the binding affinity of Lmb to laminin, implicating the role played by the metal binding residues in maintaining the correct conformation of the protein for its binding to laminin. A highly disordered loop, proposed to be crucial for metal acquisition in homologous structures, was deleted in Lmb by mutation (ΔLmb) and its crystal structure was solved at 2.6 Å. The ΔLmb structure was identical to the native Lmb structure with a bound zinc ion and exhibited laminin binding activity similar to wild type protein, suggesting that the loop might not have an important role in metal acquisition or adhesion in Lmb. Targeted mutations of histidine residues confirmed the importance of the zinc binding crevice for the structure and function of the Lmb adhesin.

## Introduction

*Streptococcus agalactiae* (Group B Streptococcus, GBS) is the leading cause of bacterial sepsis and meningitis in neonates [1]. Its colonization and invasion into the blood stream is mediated by bacterial surface adhesins, their preferred targets being human extracellular matrix proteins like laminin, fibrinogen, collagen and fibronectin. Laminin is a 900 kDa multidomain glycoprotein of the extracellular matrix and forms a major component of the basement membrane [2].

**Figure 1:**
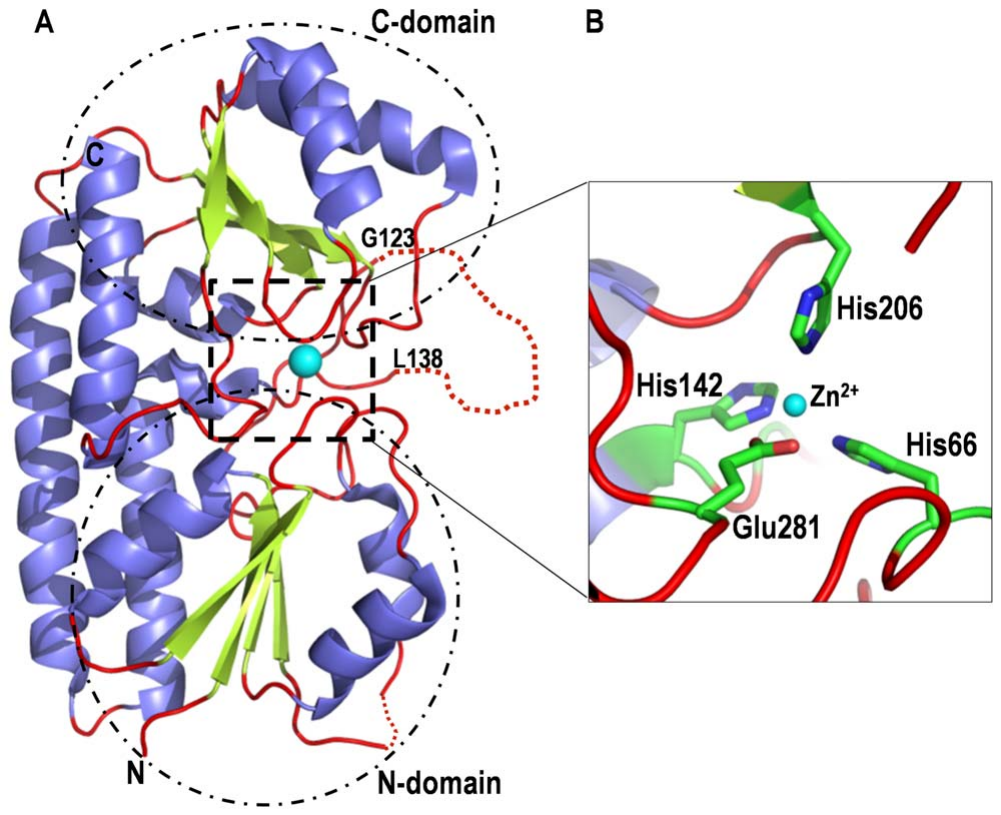
Crystal structure of Lmb of *Streptococcus agalactiae*. (**A**) Ribbon diagram of the structure of Lmb showing two domains. The metal binding site is at the interface of the two domains and the bound zinc ion is shown as cyan sphere. Near the metal binding site a long disordered loop between residues G123 and L138 is indicated by dotted lines. (**B**) Close up view of the metal binding site of Lmb. The zinc is coordinated by three histidines and a glutamate (shown as stick models).

Lmb, a laminin binding adhesin of *S. agalactiae* is a surface associated 34 kDa lipoprotein [3]. It mediates streptococcal adherence to laminin isolated from host placental membranes and is also important for the invasion of human brain endothelial cells [4]. We recently solved the structure of Lmb [5] which shows that it is a bilobed protein and shares a high degree of homology to the cluster 9 group of periplasmic solute binding proteins (SBPs). Lmb is made of two domains with (α/β)_4_ topology (Fig. 1A) and the domains are connected by a long disordered loop. Close to this loop, at the interface of the two domains, a zinc binding site is observed where the Zn^2+^ ion is coordinated by three histidines (His66, His142 and His206) and a glutamate (Glu281) (Fig. 1B).

Analysis of the structure and the genomic organization of Lmb is indicative of its role in metal binding, in addition to laminin binding. The SBPs such as ZnuA of *Escherichia coli* [6], *Salmonella enterica* [7] and Synechocystis sp. [8]–[9], TroA of *Treponema palladium* [10], [11] and AdcAII [12] and PsaA [13] of *Streptococcus pneumoniae* are encoded in an operon together with a permease, an ATPase and a regulator. Lmb and its closest homolog, Lbp of *S. pyogenes* [14], however, do not form a part of an ATPase operon but are rather co-transcribed along with a streptococcal histidine triad protein (Sht) [15]–[16]. The homologous histidine triad protein in *S. pneumoniae* (Pht) has not been functionally characterized but its structure reveals that zinc can bind to multiple HxxHxH histidine triad motifs [16]. This indicates that Lmb and Lbp, being co-transcribed with a zinc binding protein, are likely to play an important role in zinc homeostasis. Interestingly, among the SBPs characterized so far, only the streptococcal proteins PsaA [13] and Lbp [14] are known to function as adhesins as in the case of Lmb [2]. Studying the dual role played by Lmb and its homologs in adhesion and metal homeostasis can therefore provide profound insights into the function of these proteins.

To understand the correlation between laminin binding and metal binding in Lmb, we have generated single, double and triple mutants of Lmb involving the metal binding histidine residues. The solute binding proteins of the ABC zinc transport system possess a distinctive long loop close to the metal binding site. This loop is highly charged and acidic in ZnuA of *E. coli*, *S. enterica,* Synechocystis sp., [6]–[8] and uncharged as seen in Lmb, TroA, PsaA and Lbp [5], [10], [13]–[14]. The entire loop was disordered in the crystal structure of the majority of these proteins including Lmb. It was also believed that this loop may be involved in zinc binding and was called the “metal binding” loop [6]–[8]. A truncation mutant (ΔLmb) has also been made by deleting this loop (residues 123 to 138) and the interaction of all the point mutants and ΔLmb with laminin was analyzed using ELISA and dot blot assays. Further, we have determined the crystal structure of ΔLmb at 2.6 Å resolution to understand the role of this loop in the metal acquisition of Lmb. Interestingly, the ΔLmb structure shows the presence of zinc, which has not been observed in similar structures (Δ138–173 ZnuA-Syn [9] and Δ118–141 ZnuA-Se [7]) reported so far. To analyze the secondary and tertiary structural changes in Lmb mutants, CD and fluorescence spectroscopy studies have been carried out. Altogether, the mutational, structural and spectroscopic studies have provided a clear understanding of the role played by these individual amino acids and the metal binding loop in the structure and function of Lmb.

## Materials and Methods

### Bacterial strains and growth conditions

*E. coli* strains DH5α and BL21 (DE3) pLysS and their derivatives containing plasmids were grown at 37 °C in Luria-Bertani (LB) broth with agitation or on LB agar supplemented, when appropriate, with ampicillin (100 µg/ml).

### Site-directed mutagenesis

As a template for site directed mutagenesis, Lmb-His tag (wt Lmb) construct harboring the *lmb* gene (20-306), devoid of lipid anchor was used [3]. The metal binding loop of Lmb (Gly123-Leu138) was replaced by a shorter loop of 5 residues by amplifying wt Lmb using the primers 1, 2, 3 and 4 with the Expand Long Template PCR system (Roche Diagnostics GmbH, Mannheim). The PCR product was purified using Nucleospin Extract II kit (Macherey-Nagel, Düren, Germany), followed by restriction digestion with SmaI. 100 ng of the digested PCR product was ligated with pET-21a using 2.5U of T4 DNA ligase (Roche, Mannheim, Germany) according to the manufacturer’s instructions. The resulting plasmid (ΔLmb) was transformed into DH5α and BL21 (DE3) and the strain designated BSU 689.

The point mutations involving histidine residues H142A, H142-206A and H142-206-66A in the metal binding center were generated with the primers 5, 6, 7, 8, 9 and 10 using Phusion DNA polymerase (NEB). H142A mutation (strain BSU 716) was carried out using wt Lmb as the template while H142-H206A double mutant (strain BSU 718) and H142-H206-H66A triple mutant (strain BSU 778) were generated using H142A and H142-H206A plasmids respectively as the template. The PCR products were digested with DpnI for 1 h and transformed into DH5α. Colonies positive for mutation(s) were identified by sequencing. The plasmid was isolated and transformed into BL21 (DE3) pLysS.

Similarly, another three amino acids DPH(140–142) were mutated to the sequence ARD using primers 11 and 12 by amplifying the entire plasmid. The resulting plasmid was transformed into DH5α and BL21 (DE3) and designated as DPH(140–142)-ARD (BSU 691). Site directed mutagenesis of H66N was achieved by two PCR assays using primers 13, 14, 15 and 16 with wt Lmb as the template. 100 ng each of the purified PCR product was used as a template in a subsequent PCR reaction using primers 14 and 16 that contain restriction sites NdeI and XhoI respectively. The resulting PCR product was cloned into pET21a vector as described previously and transformed into BL21 (DE3) (BSU 702). Using BSU 702 as a template, mutation of DPH(140–142)-ARD+H66N was introduced in that using the primers 11 and 12 (BSU 704) as described previously.

Pro279, a residue located near the metal binding site but not directly involved in metal coordination was mutated to Ala using the primers 17 and 18 and designated BSU 777. His264 which is also not involved in metal coordination but located at a site distal from the metal binding region was mutated to Ala using the primers 19 and 20 (BSU 751). All the mutations were verified by DNA sequencing. The sequence of the primers used for the mutation experiments and the strain designations used are listed in Table 1.

**Table 1:** Primers used for deletion and site directed mutagenesis of Lmb and the strain designations.

Primer No.	Primer Sequence (5′-3′)	Strain designation	Mutation
1	ATT CCC GGG TTT GAC TCT ATC TAG TGT CAG	BSU 689	Δ(G123-L138)
2	ATA CCC GGG GCG ACA GTT TAT GAC CCA C		
3	GCC GCG CAT ATG TGT GAT AAG TCA GCAAAC CCC A		
4	GCC GCG CTC GAG CTT CAA CTG TTG ATA GAG CAC TTC C		
5	GCG ACA CTT TAT GAC CCA GCT ACC TGG ACG GAT CCC	BSU 716	H142A
6	GGG ATC CGT CCA GGT AGC TGG GTC ATA AAG TGT CGC		
7	GTG ACG CAA GCC ACG GCA TTT TCT TAT CTG	BSU 718	H142-206A
8	CAG ATA AGA AAA TGC CGT GGC TTG CGT CAC		
9	CAA TCA GGT GCA GGC ATT GCT TCC TTT GAA CCG	BSU 778	H142-206-66A
10	CGG TTC AAA GGA AGC AAT GCC TGC ACC TGA TTG		
11	ATT CCC GGG ATA CCT GGA CGG ATC CCG TTT TAG	BSU 691	DPH(140-142)-ARD
12	ATA CCC GGG CAT AAA GTG TCG CAG GGT CAA TGC		
13	ATCAGGTGCAGGCATTAATTCC	BSU 702	H66N
14	GTG GTG CTC GAG CTT CAA CTG TTG ATA GAG CAC TTC C		
15	ACG GTT CAA AGG AAT TAA TGC C		
16	GAT ATA CAT ATG TGT GAT AAG TCA GCA AAC CCC A		
11	ATT CCC GGG ATA CCT GGA CGG ATC CCG TTT TAG	BSU 704	DPH(140-142)-ARD+H66N
12	ATA CCC GGG CAT AAA GTG TCG CAG GGT CAA TGC		
17	AAA GTA AAG ACA TTA AGT GCA CTT GAA GCT GCT CCA	BSU777	P279A
18	TGG AGC AGC TTC AAG TGC ACT TAA TGT CTT TAC TTT		
19	GCT AAA GTA AAG ACA TTA AGT GCA CTT GAA GCT GCT CC	BSU 751	H264A
20	GG AGC AGC TTC AAG TGC ACT TAA TGT CTT TAC TTT AGC		

### Mutant protein expression and purification

All the mutant proteins were expressed as the insoluble fraction in the pellet with the exception of ΔLmb, H264A and P279A. Different *E. coli* host strains, including Rosetta (DE3), Rosetta gami, C43 etc. were used to solubilize them. IPTG (isopropyl β-D-1-thiogalactopyranoside) induction given at various temperatures did not increase the solubility of the protein. However, a small amount (0.2–0.5 mg/ml) of the soluble fraction of the point mutant proteins was obtained by decreasing the induction period to 2.5 h. Cell lysis and protein purification were carried out using buffer containing 50 mM NaH_2_PO_4_ pH 8.0 and 300 mM NaCl. 3 ml of the supernatant was applied to a 1 ml Ni-TED column (Machery-Nagel) and eluted with buffer containing 250 mM imidazole. The purity of the proteins was assessed on 12.5% SDS-PAGE gel and concentration of the proteins measured using UV-visible spectrophotometer at a wavelength of 280 nm.

ΔLmb was purified as described for wt Lmb for crystallization studies [17]. The concentration of the protein measured using UV-spectrophotometer at 280 nm was 40 mg/L of culture. To determine the oligomeric state of the protein in solution, gel filtration chromatography was carried out using a Superdex S-75 column (GE Biosciences).

### Crystallization

ΔLmb was crystallized using sitting drop method as used for wt Lmb [17] but with slight variations in the crystallization conditions. Initially very thin plate like crystals were obtained at 293 K by mixing 1 µl of protein solution (40 mg/ml) and 1 µl of reservoir solution containing 30% PEG2000 monomethyl ether as a precipitant. Various optimization procedures like addition of reducing agents and salts to the reservoir solution were tried to improve the size of the crystals. Finally diffraction quality crystals were obtained with 30% PEG2000 monomethyl ether, 0.1 mM sodium citrate pH 4.0 and 10% ethylene glycol and used for data collection.

### Data collection, processing and structure determination

ΔLmb crystals were directly taken from the crystallization drop in a loop and flash cooled in the liquid nitrogen stream at 100 K. Initial diffraction analysis was carried out at our in-house data collection facility. Because of the thin nature of the crystals, a good data set could not be collected at the home source. Subsequently diffraction studies were carried out at the XRD1 beamline at Elettra, Italy. A total of 120 frames were collected with an oscillation step of 1° and an exposure time of 30 seconds per frame. Diffraction images were indexed, integrated, merged and scaled using Automar software package [18]. The data collection statistics are summarized in Table 2.

**Table 2:** Statistics of diffraction data collection and structure refinement of ΔLmb.

Crystal parameters	
Space group	P2_1_
Unit cell parameters (Å, °)	a = 42.40, b = 93.99, c = 66.88; β = 105.42
Z’ (mol/asymmetric unit)	2
Matthews coefficient (Å^3^/Da)	1.89
Solvent (%)	34.90

Data processing statistics	
Temperature of measurement (K)	100
Wavelength (Å)	1.000
Resolution (Å)^a^	40 – 2.6 (2.74 –2.60)
Total reflections	42842
Unique reflections	15223
Completeness (%)	97.3 (96.6)
*R*_merge_ (%)	9.5 (43.1)
Mean I/σ(I)	6.8 (1.9)

Refinement statistics	
Resolution range (Å)	20 – 2.6
*R*_work_/*R*_free_ (%)*^b^* (All data with 0 sigma cutoff)	22.8/27.0
Number of non hydrogen protein atoms	4162
Number of water molecules	76
Number of Zn^2+^ ions	2
Root mean square deviation, bond length (Å)	0.010
Root mean square deviation, bond angle (°)	1.552
Average protein *B* factor (Å^2^)	39.43
Ramachandran plot (%)	
Preferred region/Allowed region/Outliers	94.7/4.6/0.7
PDB code	4H0F

a Parenthetical terms are for the highest resolution shell. *^b^ R*_free_ used a random 5% reserve of the working set of reflections.

### Structure determination and refinement

The structure of ΔLmb was determined using molecular replacement method with structure of wt Lmb (PDB 3HJT) as the starting model. The structure was solved using automatic structure determination software Autorickshaw [19] which identified two molecules in the crystallographic asymmetric unit. The partially refined model obtained from Autorickshaw had an R_factor_/R_free_ of 0.25/0.32 at 3.0 Å resolution. Subsequently this model was subjected to a few cycles of manual building followed by positional and individual B factor refinement. The final model had an R_factor_/R_free_  =  0.228/0.270 (at the resolution of 2.6 Å using all data with 0 σ cutoff). At this stage 2Fo-Fc and Fo-Fc maps clearly revealed an electron density connecting the residues Lys122 and Tyr139. For generating ΔLmb, the primers were designed such that five residues (PGATV) would connect Lys122 and Tyr139. The refinement statistics are summarized in Table 2.

### Dot blot assay for Lmb binding to laminin

The ability of Lmb to bind to EHS (Engelbroth-Holm-Swarm) laminin, a variant of human placental (HP) laminin was assessed by using dot blot experiments. EHS (1 mg/ml) and HP (0.5 mg/ml) laminin were purchased from Sigma-Aldrich. Equal concentrations of EHS and HP laminin (2 µl of 0.5 mg/ml) were spotted on a nitrocellulose membrane and blocked with PBST (phosphate buffered saline with 0.1% Tween 20) + 3% BSA (Bovine Serum Albumin) overnight at 277 K. The membrane was incubated with Lmb (10 µg/ml) at room temperature for 2 h, washed with PBST and incubated with anti-Lmb polyclonal antibody in PBST for 2 h. After three washes with PBST, the membrane was probed with anti-IgG-alkaline phosphatase for 2 h at room temperature. The membrane was washed thrice with PBST and the bound alkaline phosphatase was detected using BCIP/NBT substrate. Dot blot analysis was also carried out for wt Lmb, ΔLmb, DPH(140–142)-ARD, H66N, H142-206A and H142-206-66A with HP laminin using the procedure described above. For negative control, laminin was incubated with collagen binding protein, CbpA of *Arcanobacterium pyogenes* containing a His-tag and jack bean urease, devoid of any tag. 1 µl of 0.5 mg/ml of fibronectin from human plasma (Sigma-Aldrich) and collagen type VI (Sigma-Aldrich) were also used as negative controls and dot blot carried out with wt Lmb, H142-206-66A and H264A.

### Binding of Lmb and its mutants to both types of laminin

Binding of Lmb and its mutants to laminin was evaluated using enzyme-linked immunosorbent assay (ELISA) method using EHS and HP laminin. 10 µg/ml of laminin was prepared in PBS (phosphate buffered saline) buffer and coated on a 96-well plate (100 µl/well) and incubated at 310 K for 2 h. The excess unbound laminin was washed with PBST. The plate was blocked with 200 µl of 1% BSA in PBS for 2 h at 310 K. Decreasing concentrations of wt Lmb and all mutants (10 µg/ml, 5 µg/ml, 1 µg/ml, 0.5 µg/ml and 0.1 µg/ml) were added to each well and incubated at 310 K for 1.5 h. The plates were washed with PBST thrice and 100 µl of polyclonal anti-Lmb antibody diluted in PBST was added to each well and incubated at 310 K for 1.5 h. Subsequently, the plate was again washed with PBST and probed with 100 ul/well of goat anti-mouse IgG alkaline phosphatase conjugate diluted in PBST (Calbiochem). 100 µl of the substrate solution containing PNPP (1 mg/ml in 10 mM diethanolamine and 0.5 mM MgCl_2_; pH 9.8) was added to each well and absorbance read at 405 nm.

### CD spectroscopic studies

The far UV CD spectra of wt Lmb, ΔLmb, H264A, P279A, DPH(140–142)-ARD, H66N, DPH(140–142)-ARD+H66N, H142-206A and H142-206-66A were recorded using a J-720 spectropolarimeter (JASCO) in 10 mM sodium phosphate buffer, pH 7.0. The scans were taken from 190 to 240 nm at 25 °C. A rectangular cuvette of 1 mm path length was used throughout the experiment. The concentration of all the samples was kept constant at 0.5 mg/ml. Data were recorded at a scan speed of 20 nm/min with a response time of 4 s at a bandwidth of 1 nm and at 0.1 nm intervals. Triplicate scans were taken for each sample. The buffer baseline was subtracted in each case.

### Fluorescence spectroscopy

Tertiary structure analysis of Lmb and its mutants was performed using fluorescence spectroscopy. Wt Lmb, ΔLmb, H264A, P279A, DPH(140–142)-ARD, H66N, DPH(140–142)-ARD+H66N, H142-206A and H142-206-66A were studied using a Perkin-Elmer LS 45 Fluorescence Spectrometer with a 1 mm path-length quartz cell, 1 nm excitation bandwidth and 10 nm emission bandwidth. 2 µM of the protein in 50 mM sodium phosphate buffer was used. The proteins were incubated with 1-amino-2-naphthol-4-sulfonic acid (ANS) at 37°C in dark for 15 min and the change in the tertiary structure was assessed in the range of 400–700 nm with an excitation wavelength of 285 nm. Intrinsic tryptophan fluorescence was measured with an excitation wavelength set at 295 nm and emission spectra were recorded in the spectral range of 310–400 nm.

## Results

### Lmb binding to different laminin types

**Figure 2:**
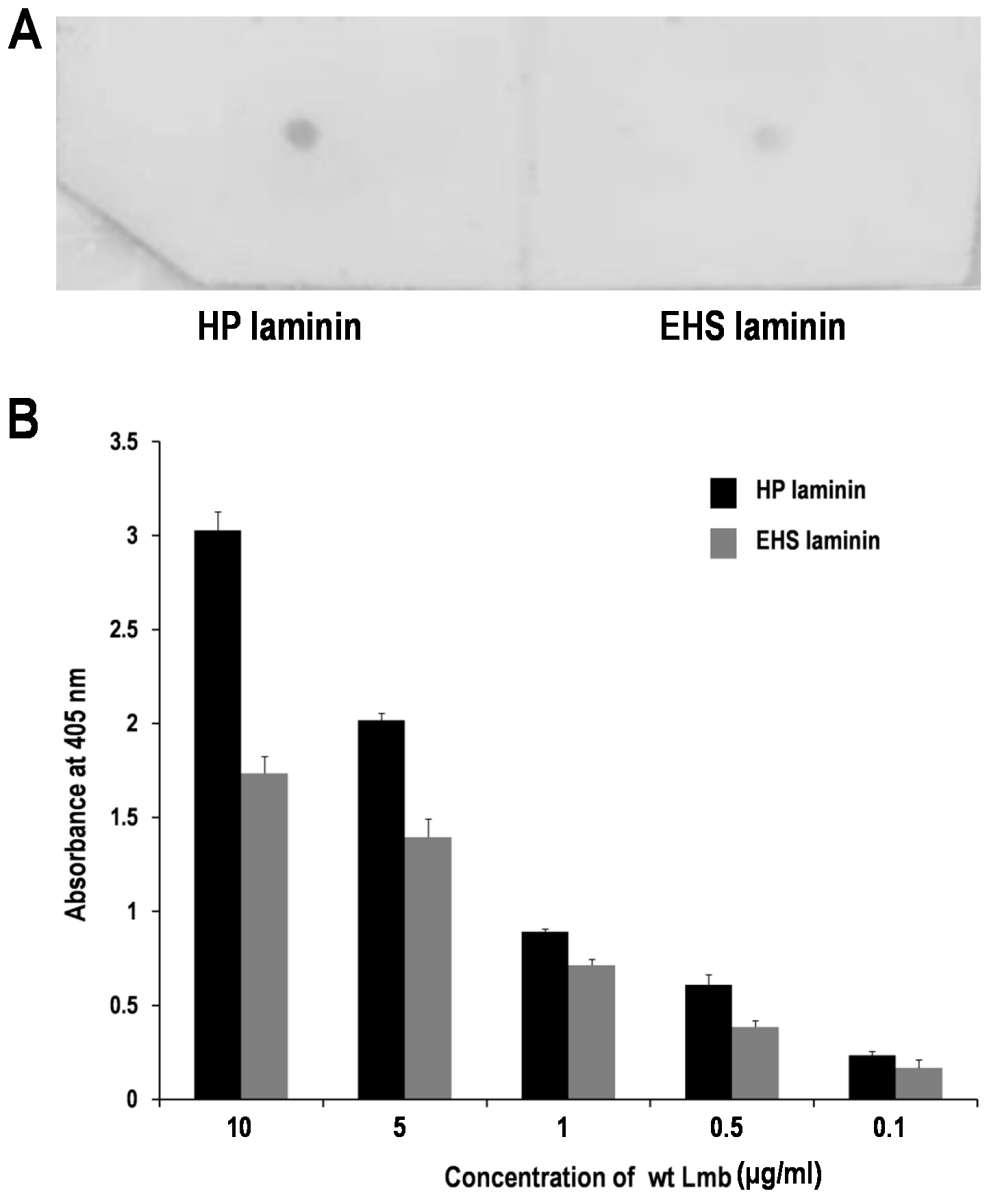
Analysis of interaction of wt Lmb with human placental (HP) and EHS (Engelbroth-Holm-Swarm) tumor laminin. (**A**) Dot blot analysis showing the interaction of wt Lmb with HP and EHS laminin. Equal concentrations (10 µg/ml) of HP and EHS laminin were spotted on a nitrocellulose membrane and probed with (10 µg/ml) wt Lmb protein. The dot blot shows that Lmb binds to both types of laminin. However, the intensity of the HP laminin spot is higher when compared with EHS Laminin spot suggesting the former has high affinity towards Lmb. (**B**) ELISA analysis showing the binding of wt Lmb to HP and EHS laminin. Different concentrations of wt Lmb was added to microtiter plates coated with 10 µg/ml of HP and EHS laminin and binding quantified at 405 nm. The values in the graph represent the mean+ standard deviation for the experiment in triplicates. This experiment clearly suggests that Lmb has higher affinity to HP laminin than EHS Laminin and thus supports the dot blot experiment.

Previous reports have demonstrated the affinity of Lmb to HP laminin [3]. Dot blot analysis has been carried out to study the interaction of Lmb with EHS laminin, another laminin variant, from mouse sarcoma. EHS and HP laminins are isotypes which share nearly the same structural organization [2]. Dot blot analysis confirms the affinity of Lmb to both types of laminin (Fig. 2A). Subsequently ELISA analysis was also carried out to ascertain the binding quantitatively. Fig. 2B shows the absorbance at 405 nm for varying concentrations of wt Lmb. The graph shows that Lmb has 30% higher affinity to HP laminin when compared to EHS laminin.

### Structure of ΔLmb and role of metal binding loop

**Figure 3:**
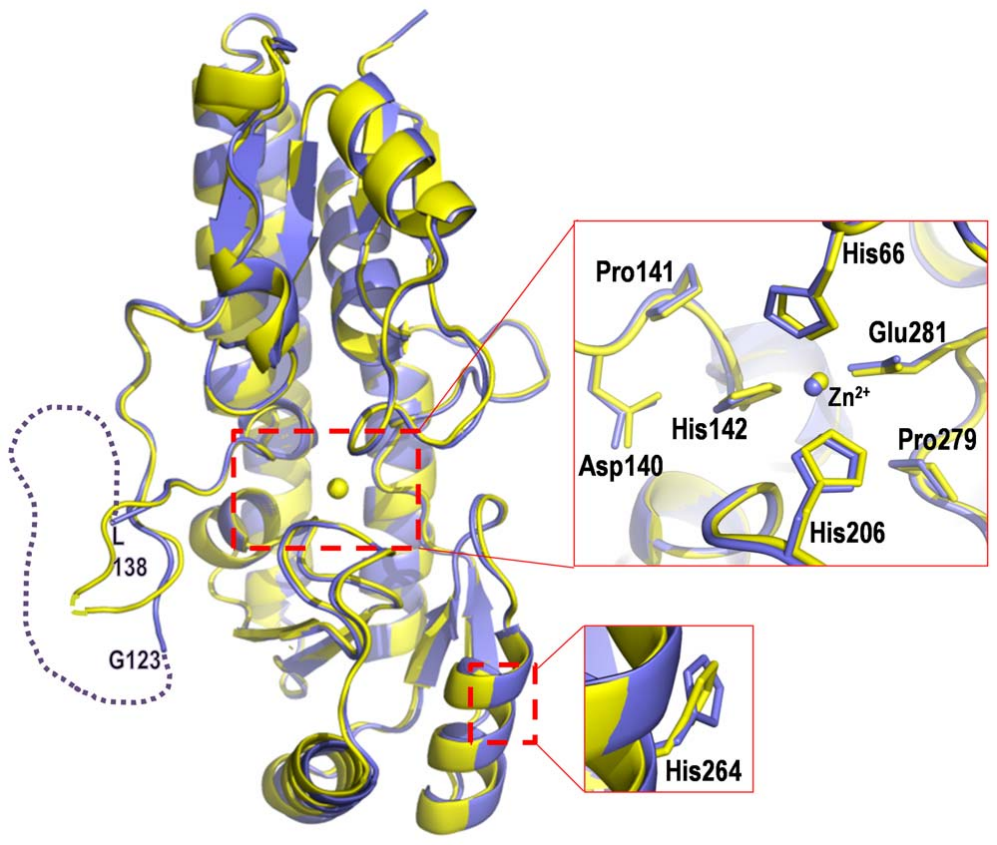
Structure of ΔLmb. Superposition diagram of wt Lmb (violet) and ΔLmb (yellow). In wt Lmb, the disordered “metal binding loop” between G123 and L138 is indicated by dotted lines. The close up view of the metal binding center is shown. The residues His66, His142, His206, Pro279, His264 and the segment DPH(140–142) were mutated in wt Lmb. The location of His264 (negative control-located away from the metal binding site) and Pro279 (negative control-located near the metal binding site) is also shown as a close up view.

In order to elucidate the role of the flexible loop (Gly123-Leu138) in metal binding and laminin binding, a construct (ΔLmb) was made, replacing the loop region with a short linker (PGATV) and its structure was determined by molecular replacement (Fig. 3) using wt Lmb as a model [5]. On comparison with wt Lmb, the backbone structure of ΔLmb including the regions at either ends of the deleted loop remains unaffected. These two structures superpose with an rms deviation of 0.4 Å for 247 Cα atoms (Fig. 3). Surprisingly, the ΔLmb structure showed the presence of bound zinc, in contrast to two other deletion mutant structures of SBPs, namely ZnuA-Se [7] and ZnuA-Syn [9], which were devoid of metal ion at the active site (Figure S1).

In the structures of ZnuA-Ec, ZnuA-Se and ZnuA-Syn a second high affinity zinc binding site has been observed and a histidine from their His-rich metal binding loop is involved in zinc ion coordination along with other histidines from the surface of the protein [6]–[8]. However, only the primary zinc binding site has been located and no such secondary site is observed in Lmb, possibly because of the absence of histidines in the loop, which cannot coordinate an additional zinc ion.

Another notable feature in ΔLmb is the packing of the two monomers in the crystallographic asymmetric unit which is different from that of the wt Lmb (Figure S2). In ΔLmb, the truncated loops of the two molecules face each other and form the interface. In contrast, in wt Lmb, the two monomers are oriented such that the loops face away from each other. A similar difference in the packing of the monomers in the crystal lattice was observed between wt and ΔZnuA-Syn [9]. It is noteworthy to mention that both wt and ΔLmb exist as a dimer in solution [5] (Figure S3), as shown by gel filtration experiments, although the physiological relevance of such a dimeric assembly is not known.

### Expression and solubility of the mutant proteins

In an attempt to evaluate the role of metal binding residues in the structure and laminin binding ability of Lmb, the metal binding histidines were mutated successively (Table 1) (Fig. 3). The mutants involving either one or all of the metal binding residues H66, H142 and H206 were observed in the insoluble fraction while ΔLmb and the negative control mutants H264A and P279A (Fig. 3) proteins were found in the soluble fraction when expressed in *E. coli*. It is evident that mutating any or all of the histidines that coordinate zinc, to Ala or Asn results in insolubility of the protein. Mutation of a histidine (H264), located away from the active site and a proline (Pro279), close to the active site did not affect the solubility of the protein. However, a small amount ∼0.2–0.5 mg/ml of the soluble fraction of the histidine mutants were obtained after optimizing the expression condition and this was used for subsequent ELISA and spectroscopic studies. The expression and purification profile of wt Lmb and all the mutants is shown in Figure S4.

### Targeted mutations of Lmb alters protein folding

**Figure 4:**
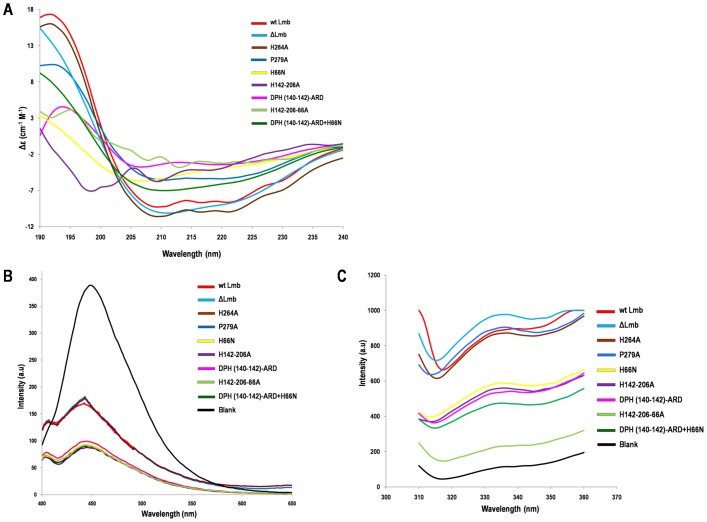
CD and fluorescence spectra of Lmb and its mutants. (**A**) The Far UV CD spectrum was recorded between 190–240 nm. ΔLmb, H264A and P279A showed same ellipticity as the wt Lmb. Significant changes in the ellipticity were observed for the mutants involving metal binding residues His66, His142 and His206 indicating these proteins have an altered secondary structure. (**B**) ANS fluorescence spectroscopy of Lmb and its mutants. Excitation wavelength was 285 nm and emission was recorded between 400 to 700 nm. Mutants of the metal binding residues show decreased fluorescence compared to wt Lmb, H264A and P279A mutant indicating they are not well folded. (**C**) Intrinsic tryptophan fluorescence of Lmb and its mutants. An excitation wavelength of 295 nm was used and emission was recorded between 310–400 nm. The spectra shows that the emission of the well folded wt Lmb, ΔLmb, H264A and P279A is higher compared to the partially folded or misfolded mutants.

The mutations involving the metal binding histidines markedly affect the secondary structure of the proteins, shown by CD spectroscopy recorded from 190 to 240 nm (Fig. 4A). The percentage of the secondary structural elements calculated from the spectra is given in Table 3. These spectra show that wt Lmb, ΔLmb, H264A and P279A (negative control) possess similar secondary structure composition. However, in other mutants, the percentage of alpha helix decreases and beta sheets increases in comparison to wt Lmb.

**Table 3:** Secondary structure composition of Lmb and its mutants obtained from CD spectroscopy.

Protein	α- helix (%)	β- sheet (%)
Wt Lmb	74.82	2.00
ΔLmb	70.82	1.24
H264A	77.22	1.67
P279A	77.16	1.51
DPH(140–142)-ARD	24.99	28.26
H66N	29.84	17.82
DPH(140–142)ARD+H66N	19.81	28.26
H142-206A	48.01	11.76
H142-206-66A	47.74	9.49

To examine whether these mutations induce a variation in the overall protein folding, fluorescence spectroscopy analysis was carried out in the presence of 1-amino naphthalene sulphonic acid (ANS). An excitation wavelength of 285 nm was used. The emission was recorded in the range of wavelengths 400 – 700 nm and a high peak at 450 nm corresponding to the dye is observed. The fluorescence intensity of wt Lmb, ΔLmb, H264A and P279A proteins were almost equal and nearly half to the intensity of dye alone. Importantly the fluorescent intensity was further decreased for the mutants H66N, DPH(140–142)-ARD, DPH(140–142)-ARD+H66N, H142A, H142-206A and H142-206-66A compared to wt Lmb (Fig. 4B). Intrinsic tryptophan fluoresence also indicated that the emission at 335 nm corresponding to wt Lmb, ΔLmb, H264A and P279A were much higher compared to the other mutants (Fig. 4C).

### Removal of zinc

Attempts were made to remove the zinc ion from wt Lmb by dialyzing the protein solution against buffer containing 1–10 mM EDTA and 1–10 mM 1, 10 phenanthroline as carried out to generate the apo-TroA of *T. pallidum* [11] and apo-ZnuA-Syn [9]. Although it was successful in those two proteins, with Lmb in all the trials (varying the concentration of metal chelators and the duration of dialysis), the protein precipitated completely, signifying that any perturbation of the metal affects the stability of the protein.

### Lmb mutants and its interaction with laminin

**Figure 5:**
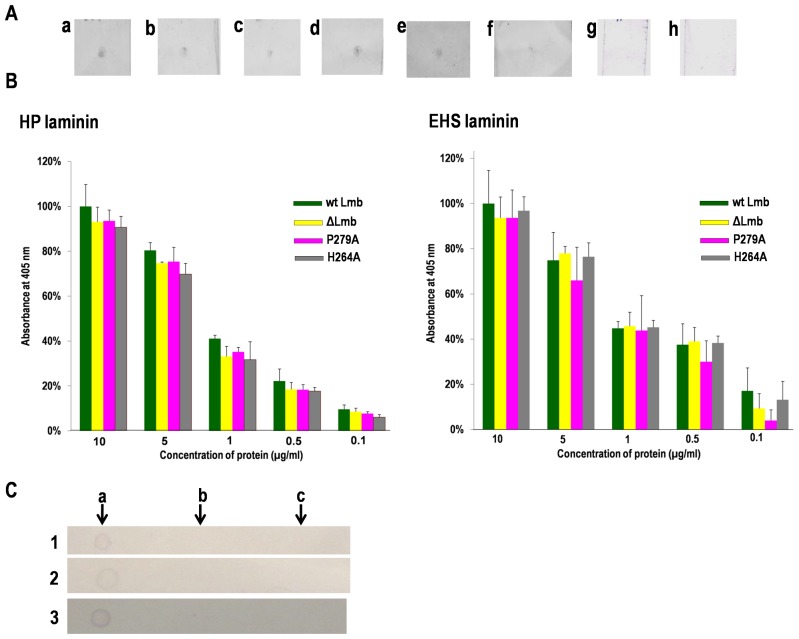
Interaction of Lmb and its mutants with laminin. (**A**) Dot blot analysis of HP laminin with (a) wt Lmb (b) DPH(140–142)-ARD (c) H66N (d) ΔLmb (e) H142-206A (f) H142-206-66A proteins (g) CbpA of *Arcanobacterium pyogenes* (negative control) (h) Jack bean urease (negative control). 10 µg/ml of laminin was spotted on the nitrocellulose membrane for binding of wt Lmb and the mutants at a concentration of 10 µg/ml. (**B**) Quantification of Lmb-laminin binding using ELISA. Lmb and its mutants were added to microtiter plate coated with HP & EHS laminin (10 µg/ml) and binding quantified at 405 nm. Data is expressed as a percentage of the binding observed for 1 µg/ml of the protein to laminin. Shown are the mean + standard deviations for the experiment in triplicates. (**C**) Dot blot analysis of (1) wt Lmb (2) H264A and (3) H142-206-66A with (a) laminin (b) fibronectin and (c) collagen. 1 µl of 0.5 mg/ml of HP laminin, fibronectin and collagen were spotted on a membrane and probed with Lmb and its mutants. Lmb shows high specificity to laminin and does not bind to fibronectin or collagen.

To further elucidate the metal binding and laminin affinity of Lmb, ELISA and dot blot analysis were carried out with the wild type and all the mutants using HP and EHS laminin. The dot blots obtained for the different mutants H142A, H142-206A, H66N etc. show that all the mutants bind to HP laminin (Fig. 5A) and EHS laminin (data not shown). Fibronectin and collagen, used as negative controls in the assay did not show binding to Lmb or the mutants.

The ELISA analysis also shows that the mutants bind to laminin thus confirming results of the dot blot analysis. Further, the ELISA analysis indicates that the wt Lmb and its mutants bind HP as well as EHS laminin in a dose dependent fashion (Fig. 5B). It is also inferred from the ELISA analysis that certain mutations significantly affect laminin binding. Specifically, the mutation involving His66 [H66N, H66A+H142A+H206A and H66N+DPH(140–142)ARD] shows significantly decreased affinity to laminin compared to the wild type protein (Data not shown). Additionally, a triple mutant DPH(140–142)-ARD was generated (Fig. 3); the DPH motif was selected for mutation because it is conserved in some proteins of the cluster 9 family and believed to form an integral part of the metal binding motif. A decrease in the affinity of DPH(140–142)-ARD mutant was observed. The protein containing both mutations, DPH(140–142)-ARD and H66N showed the least affinity to laminin, highlighting the role of H66 and the DPH motif in the function of Lmb. The decreased affinity observed might not be of significance, considering the misfolded conformation of the mutant proteins.

The binding affinity of ΔLmb was very marginally reduced, indicating that the flexible loop has no significant role in binding to laminin. Single amino acid exchanges of P279A and H264A used as negative control did not affect the binding capacity of Lmb to laminin.

## Discussion

Previous studies have demonstrated that Lmb mediates the adhesion of *S. agalactiae* to HP laminin. In this study, our data shows that Lmb also binds to EHS laminin, an isoform of the placental laminin, although with a 30% lesser affinity. This difference could be attributed to the fact that HP laminin is of human origin while EHS is murine [20]. It indicates that HP laminin is a more potent ligand for the attachment of GBS than its murine counterpart. Lmb shows no binding to fibronectin and collagen and this reveals the specificity of Lmb towards laminin (Fig. 5C). The structure of Lmb harbors a well-defined metal binding site bound to a zinc ion. To understand the function of the metal binding site in Lmb and its correlation with laminin binding, the metal binding histidine residues were mutated. We have shown, for the first time, that mutating any or all histidines at the metal binding site severely affects the solubility of Lmb, a representative of the cluster 9 family of SBPs. It is possible that the charge distribution could be affected, due to mutations of histidine, which might result in the local structural changes. This highlights the critical role played by the metal binding center in maintaining the correct protein conformation. In fact, a closer inspection of Lmb structure around the metal binding region revealed that there are hydrophobic patches (Phe39, Pro41, Trp144, Ile65, Leu91 and Pro141) and (Phe209, Leu277, Phe202, Leu212, Leu301 and Phe253) on either side of the metal binding pocket (Figure S5) and it is likely that these regions might be exposed, affecting the solubility of the protein.

Although all the metal coordinating histidine (His66, His142 and His206) mutant proteins were obtained in a soluble form in a small amount (∼0.2–0.5 mg/ml) they have a misfolded conformation. This is evidenced from the CD and fluorescence spectroscopy studies which clearly showed that all these mutants exhibit altered secondary and tertiary structures. Interestingly these mutants except H264A and P279A showed a decreased affinity to laminin compared to the wild type protein. However, the affinity seen here is probably due to a non-specific interaction between the laminin and the misfolded Lmb mutants. In contrast to the point mutants, the deletion of the disordered loop did not affect the solubility or the interaction with laminin which is evident from the binding studies where the affinity of ΔLmb towards laminin is marginally reduced in comparison to that of wt Lmb. Since the loop is highly flexible and protruding from the structure it is possible that it could make some non-specific interaction with laminin. However this loop is not very critical for laminin binding since significant differences were not observed in the affinities of wt Lmb and ΔLmb. The subsequent structural studies of ΔLmb clearly showed that it adopts a conformation as seen in the wt Lmb, with the zinc ion and identical active site geometry. Our data conclusively shows that the loop spanning residues Gly123-Leu138 do not play a significant role in metal acquisition and the interaction with laminin. It is interesting to note that the corresponding loop in both ZnuA-Syn and ZnuA-Se contain many histidines, including His60 which is involved in metal coordination. Although deletion of this loop resulted in the loss of one metal binding histidine, ΔZnuA-Se and ΔZnuA-Syn proteins folded properly, in contrast to Lmb.

The data presented here thus suggest that the role of Zn in Lmb appears to be merely a structural one in maintaining the correct fold of Lmb. Sequence comparison of Lmb with the Zn transporters such as AdcAII and ZnuA-Syn revealed that Lmb shares a low sequence identity of 30% with ZnuA-Syn and a higher identity of 65% with AdcAII. However, the electrostatic surface potential of these proteins (Figure S6) shows significant differences in the surface charge distribution, probably attributable to the laminin binding activity of Lmb. This suggests that Lmb and its close homologs might have evolved to perform other functions, namely laminin binding. Metal acquisition may represent an auxiliary function, in contrast to ZnuA proteins that have been characterized as metal transport proteins functionally.

Taken together our structural and mutational studies on Lmb strongly suggest that metal binding and laminin binding are interrelated, in the sense that metal binding maintains the protein in the proper conformation and this particular conformation is essential for the Lmb molecule to bind laminin strongly. In Lmb, any small perturbation in the metal binding site proves deleterious to its folding or correct conformation and abrogates laminin adhesion. In the case of Lbp of *S. pneumoniae*, it was proposed that its interaction with laminin was metal mediated, based on an analogy to other interacting partners of laminin [14]. Hence, it can be concluded from our studies that metal binding dictates the proper conformation and laminin binding function in Lmb.

## Supporting Information

Figure S1 **Sequence and structural comparison of Wt Lmb, ZnuA-Se and ZnuA-Syn. (A)** Sequence alignment of Lmb with ZnuA-Se and ZnuA-Syn. Sequences were aligned using MultAlin (http://multalin.toulouse.inra.fr/multalin/). Lmb shares a sequence identity of 30 and 23% respectively. The conserved residues H66, H142 and H206 and E281 coordinating zinc are highlighted in blue. The long loop positioned structurally close the metal binding site is highlighted in green. **(B)** Structure superposition of full length Lmb (blue), ZnuA-Syn (magenta) and ZnuA-Se (green). The inset shows the close up view of the disordered loops which are represented in dotted lines. The zinc ion is shown as a solid sphere. **(C)** Structure superposition of the loop truncated structures, ΔLmb (blue), ΔZnuA-Syn (magenta) and ΔZnuA-Se (green). The inset shows the close up view of the shortened loops in these structures. The zinc ion is observed only in ΔLmb.
(TIF)

Figure S2 **Difference in orientation of monomers of wt Lmb and ΔLmb in the crystal lattice.** Each molecule of wt Lmb and ΔLmb is colored yellow and green. The loop region is shown in red dotted lines. **(A)** Dimeric assembly of monomers of wt Lmb, in which the disordered loop face away from each other. **(B)** Orientation of monomers of ΔLmb showing the loops that face each other.
(TIF)

Figure S3 **Oligomeric assembly of wt Lmb and ΔLmb in solution using gel filtration chromatography.** Elution profile of the Superdex S-75 column loaded with 10 mg/ml of wt Lmb (red), ΔLmb (blue) and 10 mg/ml of BSA and 10 mg/ml of ovalbumin (red dotted lines). The latter two proteins were used as molecular weight standards. The two peaks of wt Lmb and ΔLmb correspond to the monomer (32, 29 kDa respectively) and dimeric (64, 58 kDa respectively) state of the protein.
(TIF)

Figure S4 **Purification profile of Lmb and its mutants.** A 15% SDS-PAGE gel showing purified Lmb and its mutants. The proteins were purified from 500 ml culture using Ni-NTA columns and 10 µl of purified protein was loaded in each lane. The gel picture shows that the expression of wt Lmb, ΔLmb, H264A and P279A is much higher compared to the other mutants involving one or more metal binding histidines.
(TIF)

Figure S5 **Hydrophobic patches near the metal binding site of Lmb.** Ribbon representation of Lmb showing two hydrophobic patches near metal binding center. These regions might be exposed while mutating the metal binding histidines, resulting in structural deformation, which is likely reflected in the fluorescence studies.
(TIF)

Figure S6 **Comparative electrostatic surface representation of Lmb, AdcAII and ZnuA-Syn in three different orientations.** The orientation of the molecule is shown in ribbon diagram. Significant differences were observed in the surface characteristics of these proteins and probably attributed to their different functions. Surface representation, colored according to electrostatic potential. The figure was prepared using the automated module ‘Protein contact potential’ of PyMOL. Negative potential (–77 kT/e) are red, positive potential (77 kT/e) are blue and neutral potential are white.
(TIF)
