# Supplementary figures and images for: Metal Binding Is Critical for the Folding and Function of Laminin Binding Protein, Lmb of Streptococcus agalactiae
Source: PLoS One. 2013 Jun 24;8(6):e67517. doi: 10.1371/journal.pone.0067517 (PMC3691195; doi:10.1371/journal.pone.0067517)

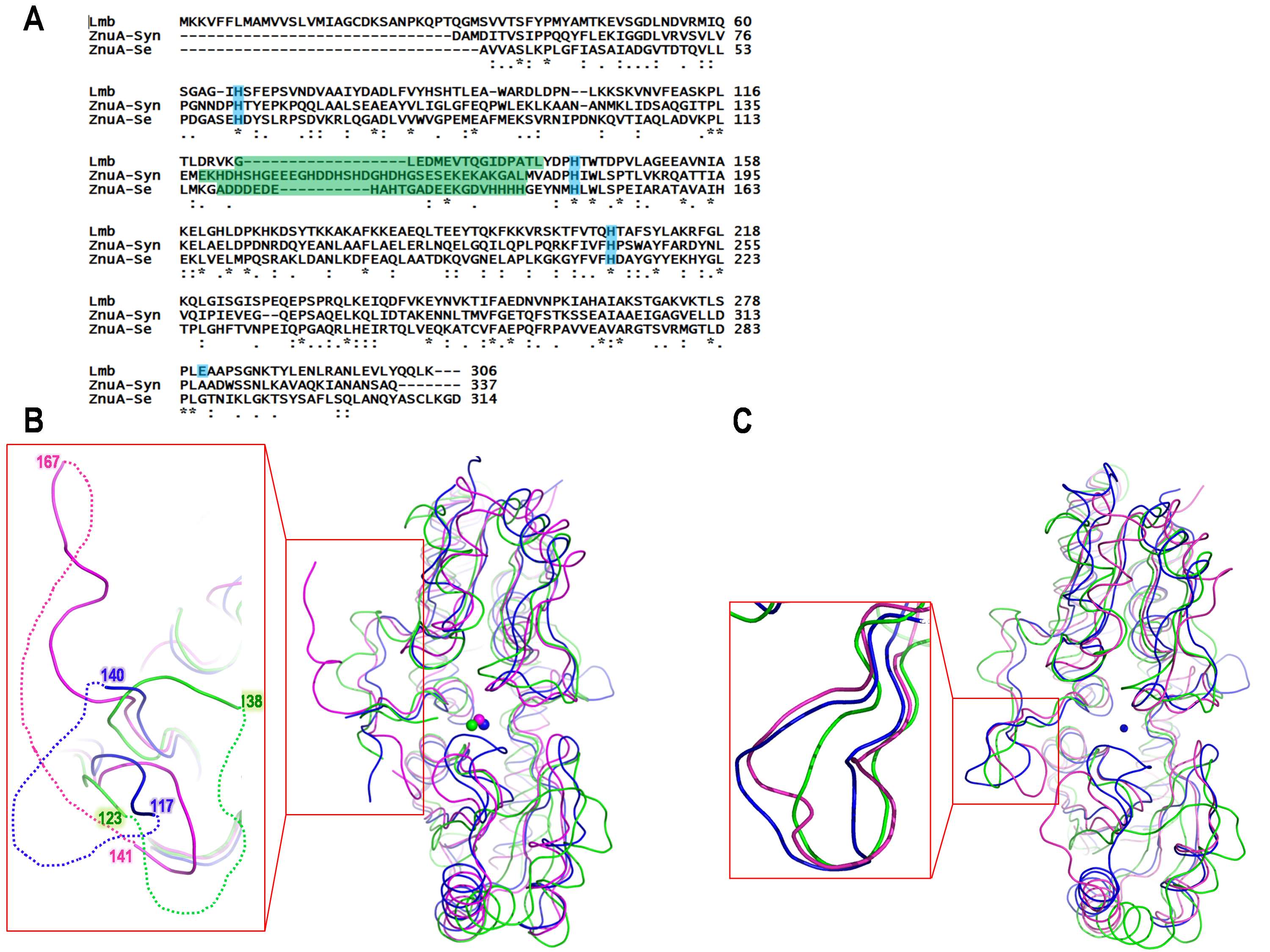

Supplement: Figure S1 — Sequence and structural comparison of Wt Lmb, ZnuA-Se and ZnuA-Syn. (A) Sequence alignment of Lmb with ZnuA-Se and ZnuA-Syn. Sequences were aligned using MultAlin (http://multalin.toulouse.inra.fr/multalin/). Lmb shares a sequence identity of 30 and 23% respectively. The conserved residues H66, H142 and H206 and E281 coordinating zinc are highlighted in blue. The long loop positioned structurally close the metal binding site is highlighted in green. (B) Structure superposition of full length Lmb (blue), ZnuA-Syn (magenta) and ZnuA-Se (green). The inset shows the close up view of the disordered loops which are represented in dotted lines. The zinc ion is shown as a solid sphere. (C) Structure superposition of the loop truncated structures, ΔLmb (blue), ΔZnuA-Syn (magenta) and ΔZnuA-Se (green). The inset shows the close up view of the shortened loops in these structures. The zinc ion is observed only in ΔLmb. (TIF) [file pone.0067517.s001.tif]

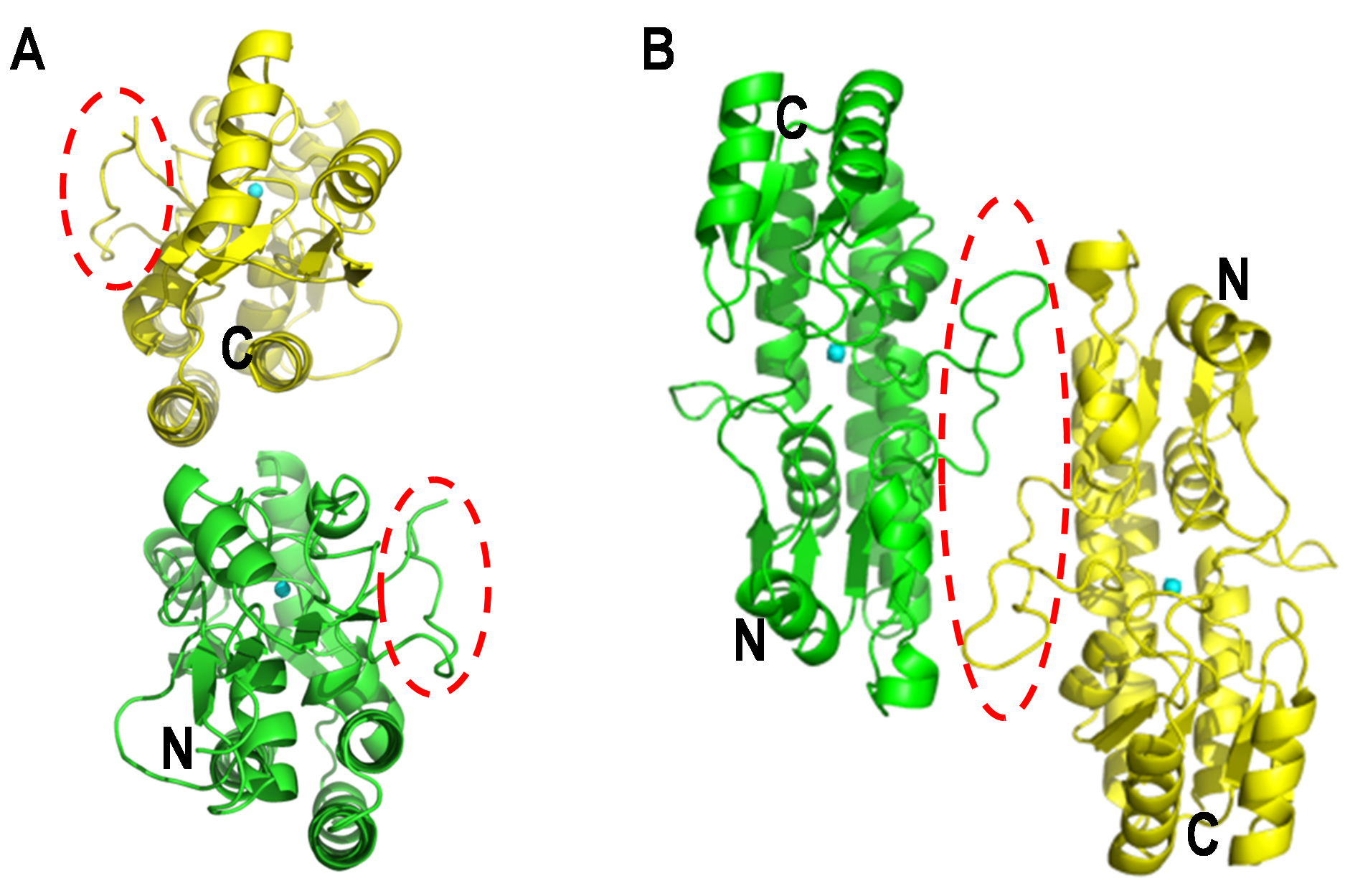

Supplement: Figure S2 — Difference in orientation of monomers of wt Lmb and ΔLmb in the crystal lattice. Each molecule of wt Lmb and ΔLmb is colored yellow and green. The loop region is shown in red dotted lines. (A) Dimeric assembly of monomers of wt Lmb, in which the disordered loop face away from each other. (B) Orientation of monomers of ΔLmb showing the loops that face each other. (TIF) [file pone.0067517.s002.tif]

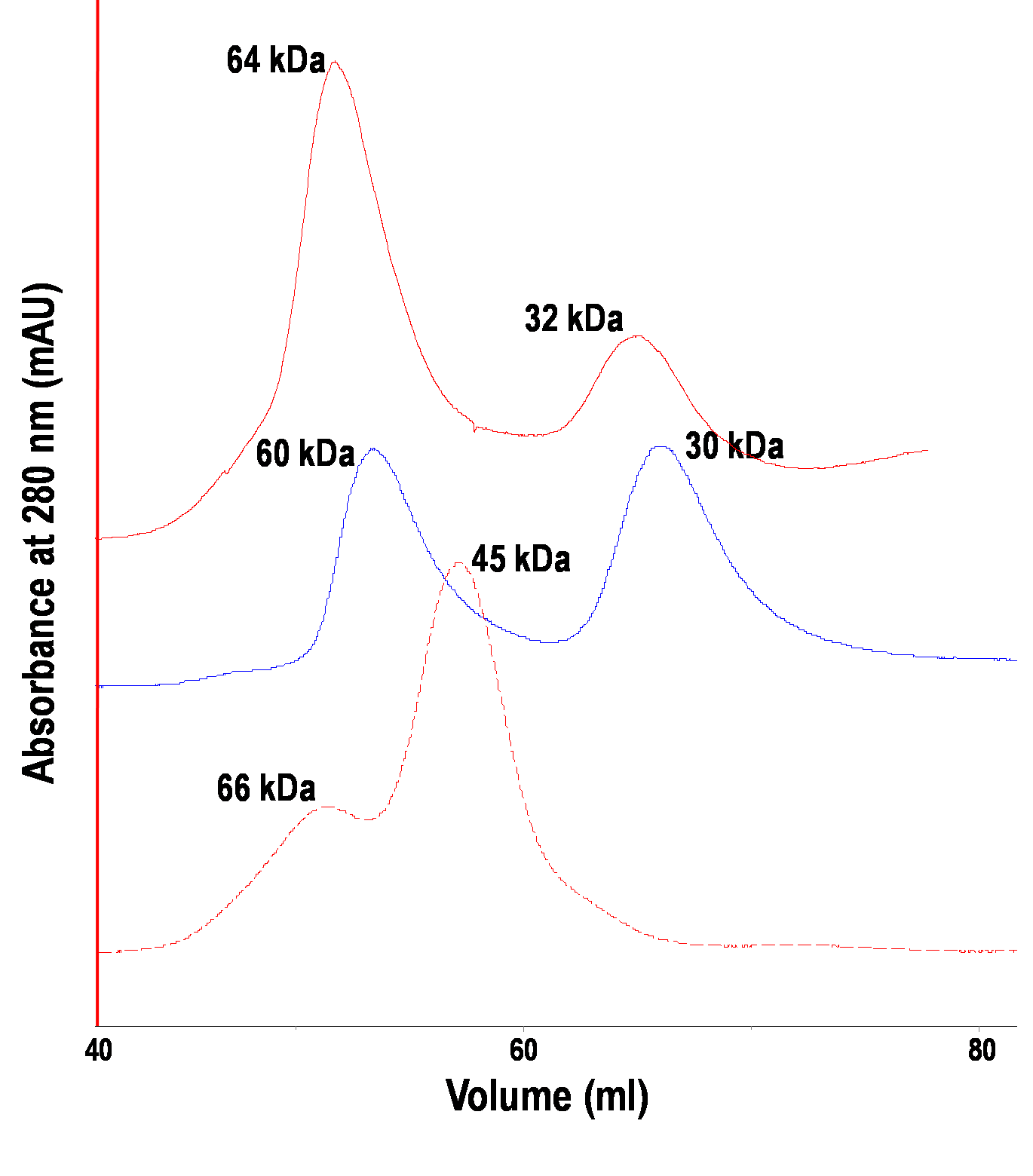

Supplement: Figure S3 — Oligomeric assembly of wt Lmb and ΔLmb in solution using gel filtration chromatography. Elution profile of the Superdex S-75 column loaded with 10 mg/ml of wt Lmb (red), ΔLmb (blue) and 10 mg/ml of BSA and 10 mg/ml of ovalbumin (red dotted lines). The latter two proteins were used as molecular weight standards. The two peaks of wt Lmb and ΔLmb correspond to the monomer (32, 29 kDa respectively) and dimeric (64, 58 kDa respectively) state of the protein. (TIF) [file pone.0067517.s003.tif]

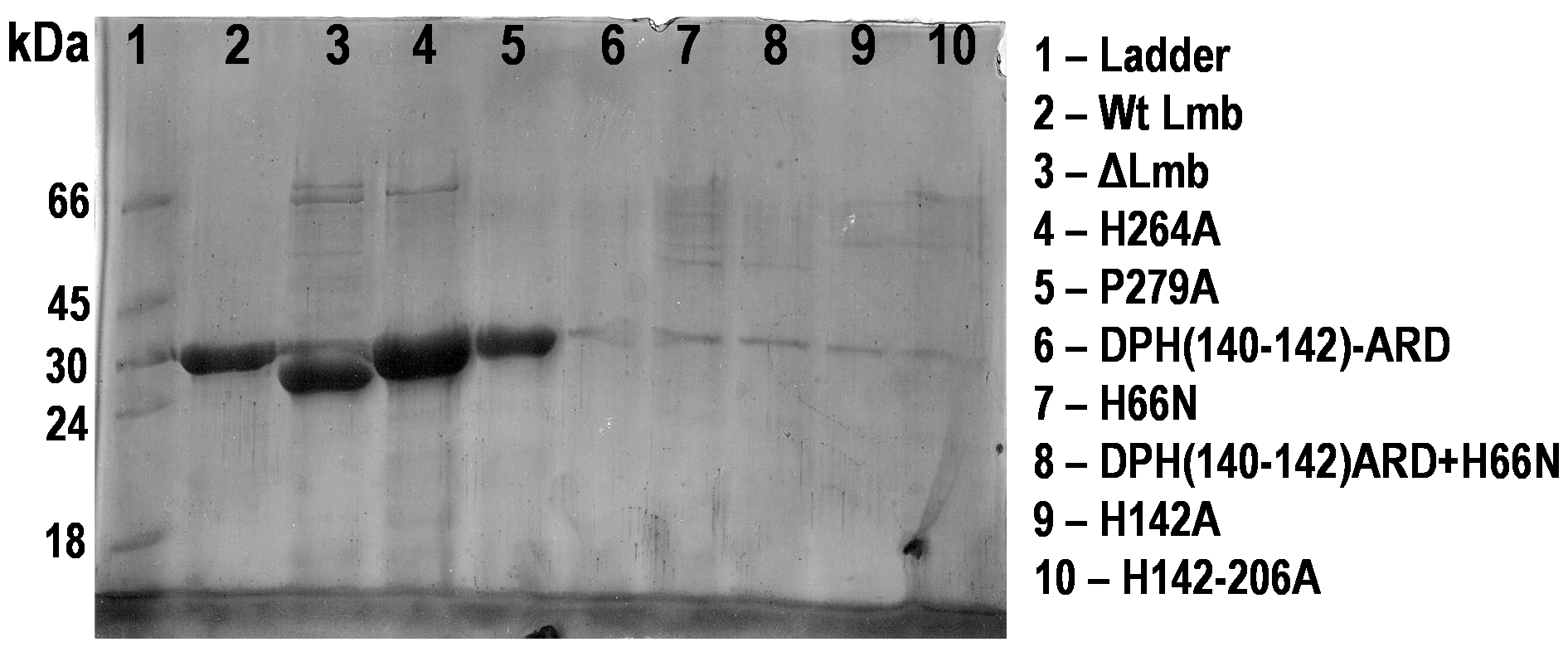

Supplement: Figure S4 — Purification profile of Lmb and its mutants. A 15% SDS-PAGE gel showing purified Lmb and its mutants. The proteins were purified from 500 ml culture using Ni-NTA columns and 10 µl of purified protein was loaded in each lane. The gel picture shows that the expression of wt Lmb, ΔLmb, H264A and P279A is much higher compared to the other mutants involving one or more metal binding histidines. (TIF) [file pone.0067517.s004.tif]

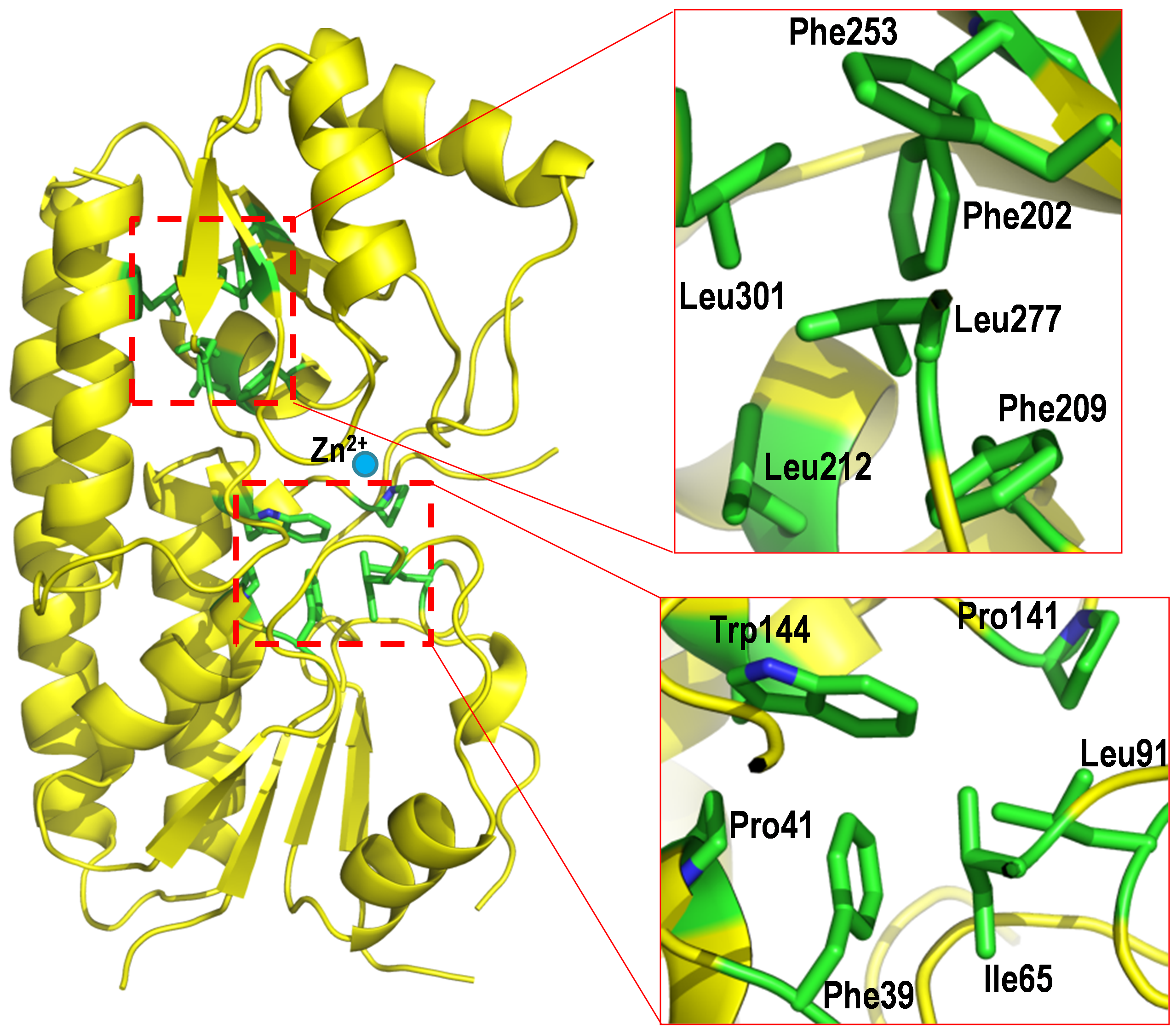

Supplement: Figure S5 — Hydrophobic patches near the metal binding site of Lmb. Ribbon representation of Lmb showing two hydrophobic patches near metal binding center. These regions might be exposed while mutating the metal binding histidines, resulting in structural deformation, which is likely reflected in the fluorescence studies. (TIF) [file pone.0067517.s005.tif]

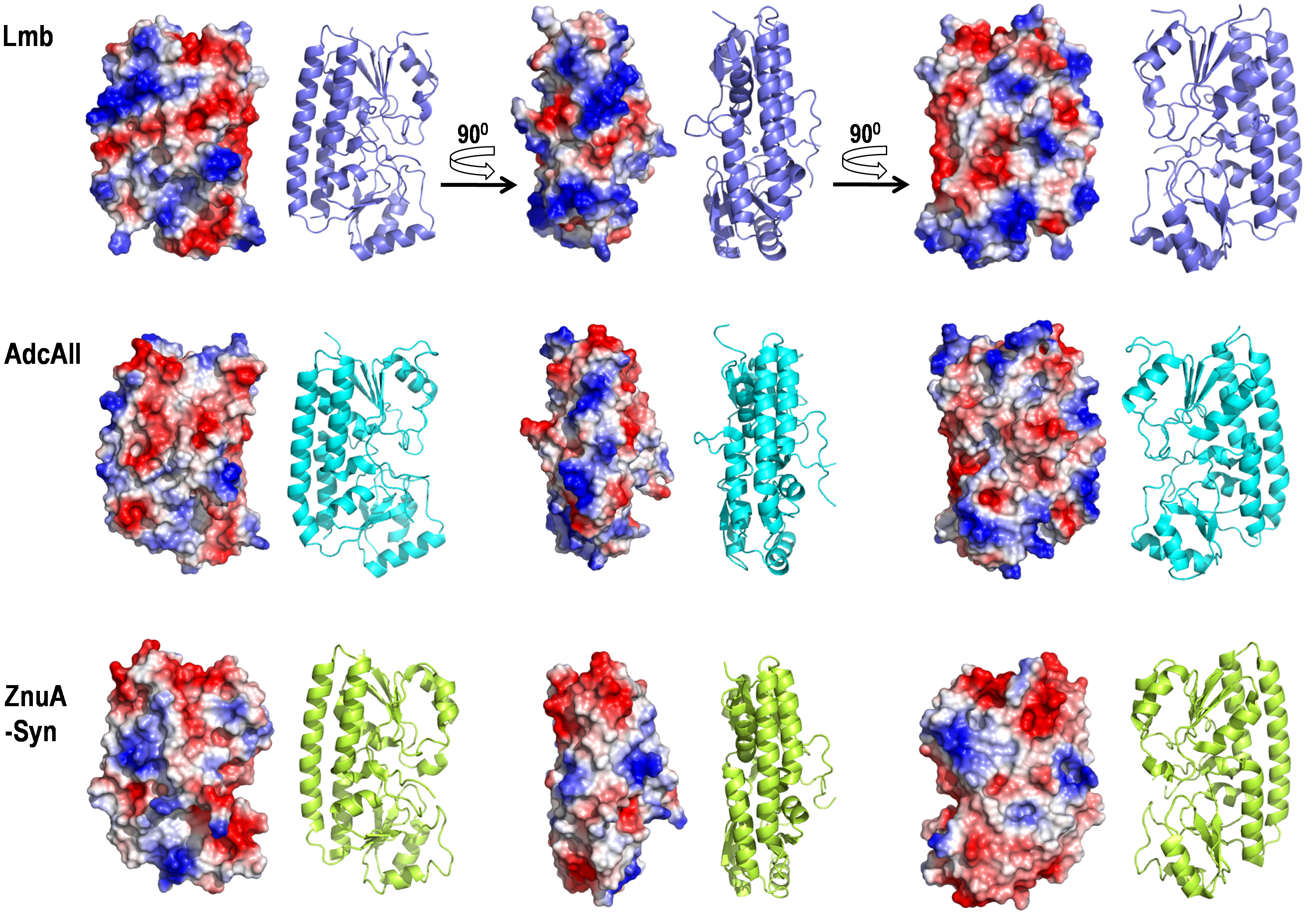

Supplement: Figure S6 — Comparative electrostatic surface representation of Lmb, AdcAII and ZnuA-Syn in three different orientations. The orientation of the molecule is shown in ribbon diagram. Significant differences were observed in the surface characteristics of these proteins and probably attributed to their different functions. Surface representation, colored according to electrostatic potential. The figure was prepared using the automated module ‘Protein contact potential’ of PyMOL. Negative potential (–77 kT/e) are red, positive potential (77 kT/e) are blue and neutral potential are white. (TIF) [file pone.0067517.s006.tif]
